# Identification and Verification of Anoikis‐related Genes in Epilepsy Through Bioinformatics Analysis

**DOI:** 10.1002/brb3.71273

**Published:** 2026-02-16

**Authors:** Haoxuan Zeng, Yanling Yuan, Tian Tan, Wenbing Zhou, Xianju Zhou, Xinhao Chen

**Affiliations:** ^1^ Department of Neurology Southern Medical University Hospital of Integrated Traditional Chinese and Western Medicine Southern Medical University Guangzhou China; ^2^ Department of Neurology Houjie Hosptial and Clinical College of Guangdong Medical University Dongguan Guangdong China; ^3^ Department of Neurology Dongguan Eighth People's Hospital Dongguan Guandong China

**Keywords:** bioinformatics, epilepsy, anoikis, GEO database, machine learning

## Abstract

**Introduction:**

Epilepsy is a significant neurological disorder characterized by a complex etiology. Understanding the molecular mechanisms, notably those implicated in the immune system and anoikis, is crucial for developing targeted therapies against epilepsy.

**Methods:**

First, two epilepsy‐related transcriptomic datasets (GSE143272 and GSE4290) were selected from the GEO database; then, the differentially expressed genes (DEGs) were identified. Focusing on anoikis in epilepsy, we screened out anoikis‐related genes (ARGs). Bioinformatics analysis and machine learning were employed for comprehensive analysis, and the research results were validated in an epileptic mouse model.

**Results:**

A total of 3525 DEGs from the GSE143272 and GSE4290 datasets were identified, and a total of 24 ARGs were obtained. The five key differentially expressed ARGs (DE‐ARGs) were screened through machine learning analysis, including ANKRD13C, PIK3R1, BSG, CEACAM6, and BRMS1; these DE‐ARGs emerged as potential biomarkers for epilepsy and were involved in various signaling pathways and immune cell activities, and results were further experimentally validated. Besides, the risk score model based on the DE‐ARGs demonstrated high diagnostic efficiency; moreover, connectivity map database analysis suggested MPEP, LY‐341495, and MDL‐28170 as potential therapeutic agents.

**Conclusions:**

This study identified the five ARGs as potential therapeutic targets, highlighting the role of anoikis in epilepsy pathogenesis. Our result provides a novel insight into the molecular landscape of epilepsy and paves the way for further exploration and the development of more effective treatment strategies.

## Graphical abstract

A total of 3525 differentially expressed genes (DEGs) from two epilepsy‐related transcriptomic datasets (GSE143272 and GSE4290) were screened, and a further 24 anoikis‐related genes (ARGs) were obtained. The five key differentially expressed ARGs (DE‐ARGs), including ANKRD13C, PIK3R1, BSG, CEACAM6, and BRMS1, were identified through machine learning (LASSO regression and WGCNA analysis).

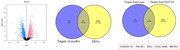

## 1 Introduction

Epilepsy, a prevalent chronic disorder of the nervous system, ranks as the third most frequent chronic brain disease. Most intractable epilepsy cases, approximately 70%, are attributed to temporal lobe epilepsy (Kalilani et al. 2018; Fiest et al. 2017; Thurman et al. 2018). Epilepsy typically involves hippocampal sclerosis, characterized by specific neuronal loss, persistent neuroinflammation, glial cell proliferation, and atypical synaptic reorganization (Aronica et al. 2010). In animal models, epileptogenic alterations primarily occur in the initial phase following an epileptogenic brain injury (Korotkov et al. 2020). Currently, no established preventive strategies or effective treatments for these alterations exist. Previous research indicates that epilepsy can trigger an immune response, leading to neuronal damage and initiating tissue repair processes (Meldrum 1990). Furthermore, neuronal loss may disrupt immune balance after epileptic seizures (Thijs et al. 2019). Epilepsy is often associated with changes in various immune cells, such as B cells, T cells, natural killer (NK) T cells, gamma delta T cells, Tregs, macrophages, mast cells, neutrophils, and monocytes (Bauer et al. 2012). However, the precise immune mechanisms underlying epilepsy remain largely unexplored. The discovery of novel characteristic genes might offer new insights into the etiology of epilepsy and potential therapeutic targets.

Anoikis, a specialized type of programmed cell death, represents a form of apoptosis occurring in the event of cells detaching from the appropriate extracellular matrix, disrupting integrin‐mediated adhesions. This prevents aberrantly proliferating cells from adhering to or growing within an unsuitable matrix (Gilmore 2005). Anoikis is marked by inhibited anchorage‐dependent growth and epithelial‐mesenchymal transition, signifying its importance not only in maintaining tissue equilibrium and development but also in regulating major conditions such as metastatic cancer, cardiovascular diseases, and diabetes (Taddei et al. 2012). Research has elucidated various elements associated with anoikis mechanisms, including TGF‐β, E‐cadherin, integrins, nuclear factor (NF)‐κB, EGFR, IGFR, Trk receptors, eEF‐2 kinase, the Hippo signaling pathway, conditions of hypoxia and acidosis, reactive oxygen species (ROS), Helicobacter pylori, and protective autophagy (Adeshakin et al. 2021). The occurrence of epileptic seizures and the subsequent neuroinflammatory response in TLE exacerbate cell and tissue damage, leading to apoptosis primarily caused by oxidative stress and excitatory amino acid toxicity due to hypoxia. This damage primarily affects glial cells, neurons, and vascular endothelial cells (Gloor and Fariello 1988). The pathological progression is characterized by a significant release of chemokines, cell adhesion molecules, and inflammatory cytokines (Vezzani et al. 2011). Considering these factors, anoikis may have a pivotal role in the development and progression of epilepsy.

In this study, we utilized the GEO database to identify variations in the expression of anoikis‐related genes (ARGs) associated with epilepsy. Our methodology involved functional enrichment analysis, consensus clustering, and immune infiltration analysis of epilepsy samples, focusing on differentially expressed genes (DEGs) associated with anoikis. Furthermore, the differentially expressed ARGs (DE‐ARGs) were identified using two different machine learning algorithms. Additionally, an epilepticus mouse model was established for validation of these results in vivo. This study aims to indicate the effectiveness of DE‐ARGs as biomarkers for epilepsy, and our results present novel insights for epilepsy diagnosis and potential therapeutic interventions.

## 2 Materials and Methods

### 2.1 Data Acquisition and Preprocessing

The gene expression data for this study were acquired from the GEO database, including GSE143272 and GSE4290 (Rawat et al. 2020; Sun et al. 2006). The GSE143272 dataset contains 50 healthy participants (the control group) and 91 epilepsy patients (the epilepsy group). The GSE4290 contains 4 control participants (the control group) and 23 epilepsy patients (the epilepsy group). After annotating these files, the R “limma” software package was utilized to identify differential expression genes (DEGs) between two groups and filter with the condition Log2|*FC*| > 1 and *p*‐value < 0.05 (Becht et al. 2016). Then, volcano plots and heatmaps were used to visually depict these DEGs. The GSE143272 dataset served as the training set, whereas GSE4290 served as the validation set.

### 2.2 GO and KEGG Function Enrichment Analyses

GO and KEGG databases are critical tools in biological research. By combining GO and KEGG enrichment analyses, researchers can quickly understand the biological functions of targets. It is important to further comprehensively explore the functions of DEGs, which are imported into the R “ClusterProfiler” software package for GO and KEGG analysis (Huang et al. 2009; Zhou et al. 2019). GO analysis includes biological processes (BP), cellular components (CC), and molecular functions (MF), meanwhile using KEGG analysis to better understand the pathogenesis of epilepsy. Finally, rank all enrichment analysis results and display the highest‐scoring terms.

### 2.3 Prediction Model Construction

The candidate gene set was selected to construct the predictive correlation model through Least Absolute Shrinkage and Selection Operator (LASSO) regression (Zhang et al. 2020). Following the integration of the expression value of individual genes, a risk score formula was established for all patients, and the weights were determined by the estimated regression coefficients from the regression analysis. Then, the score for every patient was calculated according to the risk score formula, and the accuracy of the model was assessed using ROC curves.

### 2.4 WGCNA Analysis

The WGCNA analysis can facilitate the identification of co‐expressed gene modules and screen the core modules and key targets. In this study, the R “WGCNA” software package was employed to establish the co‐expression network encompassing all genes in the dataset (Rezaei et al. 2022). The top 50% DEGs with the most significant differential folds were grouped into subgroups based on their different expression patterns, the weighted adjacency matrix was transformed into a topological overlap matrix to assess network connectivity, and hierarchical clustering was employed to delineate the cluster tree structure to find the core modules. Each module is represented by a distinct color.

We followed the official WGCNA guidelines and first assessed the impact of various candidate soft thresholds (β = 1–20) on the network's scale‐free topology. As *β* increases, *R* ^2^ begins to plateau at *β* ≈ 5, while the network's average connectivity remains at a relatively reasonable level (see Supplemental Materials Figure S1). Therefore, we selected *β* = 5 as the final soft threshold for this study, balancing the approach to a scale‐free topology and maintaining network connectivity.

### 2.5 Immune Cell Infiltration Analysis

The CIBERSORT method is commonly utilized for evaluating immune cell types in a microenvironment (Wang et al. 2022). Grounded in the principles of supporting vector regression, this method employs deconvolution analysis of the expression matrix of immune cell subtypes. The matrix encompasses 547 biomarkers that distinguish 22 human immune cell phenotypes, comprising B cells, T cells, plasma cells, and myeloid cell subsets. In this research, the CIBERSORT algorithm was employed to examine patient data, enabling the inference of the relative proportions of the 22 types of immune‐infiltrating cells. A Spearman correlation analysis was performed on gene expression and immune cell content.

### 2.6 GSEA and GSVA Analysis

GSEA is a widely used method to investigate the intricate interaction between disease classification and biological relevance (Yu et al. 2022). GSEA was applied to delve deeper into the disparities in signaling pathways between groups with high and low expression. The background gene sets utilized were annotated gene sets downloaded from v7.0 of the MsigDB, serving as annotation gene sets for subtype pathways. Subsequently, differential expression analysis of pathways between subtypes was executed, and gene sets that exhibited significant enrichment (*p* < 0.05) were ranked by consistency scores.

GSVA, a nonparametric and unsupervised method, is commonly employed to assess transcriptome gene set enrichment (Guo et al. 2023). It scores the gene set of interest comprehensively, converting gene‐level alterations into pathway‐level changes, thus helping the evaluation of biological function within the sample. Herein, the molecular signatures database was accessed to acquire the gene sets, and the GSVA algorithm was applied to generate comprehensive scores for each gene set, facilitating the examination of potential alterations in biological functionality across distinct samples.

### 2.7 Single‐cell RNA Sequencing (scRNA‐seq)

The R package “Seurat” was used to analyze the scRNA‐seq dataset (GSE190452), which contains four normal and temporal lobe epilepsy samples, respectively, including PCA and UMAP analyses. Gene expression normalization was performed utilizing the “LogNormalize” method, followed by the identification of highly variable genes utilizing the “vst” method for each sample. Significant principal components were determined via PCA, and the “JackStraw” and “ScoreJackStraw” methods were utilized for the visualization of their *p*‐value distribution. Cells can be categorized into eight distinct clusters via the “FindClusters” function. DEGs within each cluster were identified using the “FindAllMarkers” function, employing a logarithmic fold change (logFC) threshold of 0.25. Cell types were discerned per the DEGs within each cluster. The “GOplot” package was utilized to generate volcano plots for the DEGs in every cluster.

### 2.8 Animal

SPF grade C57BL/6 male mice (weighing 22–28 g) were obtained from Spebo Biotechnology Co., Ltd., Beijing, China. The animals were kept under controlled conditions at 25°C, with 60% humidity under a 12‐h light‐dark cycle, and were allowed unrestricted access to water and food. To develop the epilepsy model, pilocarpine (300 mg/kg, Sigma) was administered to the mice via intraperitoneal injection. Methyl scopolamine (2 mL/kg, Sigma) was administered for 30 min prior to pilocarpine to mitigate its peripheral cholinergic effects. The onset and severity of seizures were monitored using the modified Racine scale (Racine 1972), and studies proceeded only with mice exhibiting epilepsy. For seizure management, diazepam (4 mg/kg, Sigma) was administered intraperitoneally for 2 h after epilepsy onset (Zou et al. 2023). Subsequently, the mice were euthanized, and their hippocampal tissues were harvested for subsequent examination. All animal experiment protocols comply with local relevant laws and regulations, which have been approved by the Institutional Animal Care and Use Committee of the Southern Medical University (Grant No. 20210405006).

### 2.9 Quantitative Real‐Time PCR (qRT‐PCR)

Animal hippocampal tissue was collected. Following the manufacturer's instructions, RNA was extracted using an RNA extraction kit. After verifying the RNA purity by calculating the OD260/OD280 ratio with a NanoDrop instrument, cDNA was prepared from RNA using the PrimeScript RT Kit (R323, Vazyme). The reaction program was set as incubated at 37°C for 15 min and subsequently rapidly heated to 85°C for 5 s. Finally, the qRT‐PCR assay was performed using the SYBR qPCR Master Mix kit (Vazyme), with the program set as initial denaturation at 95°C for 30 s, with a subsequent denaturation at 95°C for 10 s, and annealing at 60°C for 30 s over 40 cycles. All specific primer information and sequences are listed in Supplementary Materials Table S1. Results were normalized to β‐actin as an internal reference in this study, and the relative expression levels of genes were calculated using the 2‐^ΔΔCt^ method (*n* = 3).

### 2.10 Western Blotting Analysis

Animal hippocampal tissue was collected. After tissue homogenization, the tissue homogenate was collected and added to RIPA lysis buffer for lysis on ice for 5 min, followed by centrifugation at 4°C and 17,400 g for 15 min to collect the protein supernatant. The protein concentration was then detected using the BCA method and normalized with RIPA. An appropriate amount of loading buffer was added to each group's protein supernatant and mixed evenly, followed by heating the protein at 100°C. The proteins were then separated by electrophoresis on a PAGE gel, with conditions set at 80 V for 30 min and 120 V for 1 h. Then, the proteins were transferred onto a PVDF membrane, with transfer conditions set at 260 mA for 2 h. After blocking with skim goat milk, the membrane was incubated with the primary antibody overnight at 4°C. It was then washed thoroughly three times with TBST and incubated with the secondary antibody at room temperature for 2 h. After another three washes with TBST, the membrane was developed using an ECL developing solution. All antibody information and dilution concentrations are listed in Supplementary Materials Table S2. All results were analyzed using ImageJ software to calculate the gray value of protein bands, with β‐actin used as an internal reference to calculate the relative protein expression levels.

### 2.11 Transcriptional Regulation Analysis of DE‐ARGs

The R “RcisTarget” was used for the prediction of transcription factors. All computations conducted by RcisTarget relied on motifs. The normalized enrichment score (NES) for motifs was contingent upon the total number of motifs in the database. Besides the motifs annotated by the source data, additional annotation files were inferred using motif similarity and gene sequences. The initial assessment of motif overexpression in the gene set involved computing the area under the curve (AUC) for each motif‐motif pair. This was determined by the recovery curve of the sequence based on the gene set. The NES for each motif was derived from the AUC distribution of all motifs in the gene set.

### 2.12 Drug Prediction Using the Connectivity Map (CMap) Database

The CMap, developed by the Broad Institute, is a database comprising gene expression profiles resulting from gene expression interventions (Fan et al. 2023). It is primarily utilized to reveal functional associations among small‐molecule compounds, genes, and disease states. The database encompasses gene chip data collected pre‐ and post‐treatment with 1309 small‐molecule drugs across five human cell lines. Treatment parameters vary, encompassing diverse drugs, treatment durations, concentrations, and other parameters. In this research, disease‐associated DEGs were leveraged to predict the targeted therapeutic drugs for the disease.

### 2.13 Statistical Analysis

All results were analyzed statistically using GraphPad Prism Software. *T*‐tests and one‐way analysis of variance (ANOVA) were used to check for statistical significance. Differences between the two groups were measured by t‐test, and comparisons between the groups were evaluated by ANOVA. Data were expressed as mean ± SD, and *p* < 0.05 is considered to indicate a significant difference.

## 3 Results

### 3.1 Identification of DEGs in Samples Between Epilepsy and Normal

**FIGURE 1:**
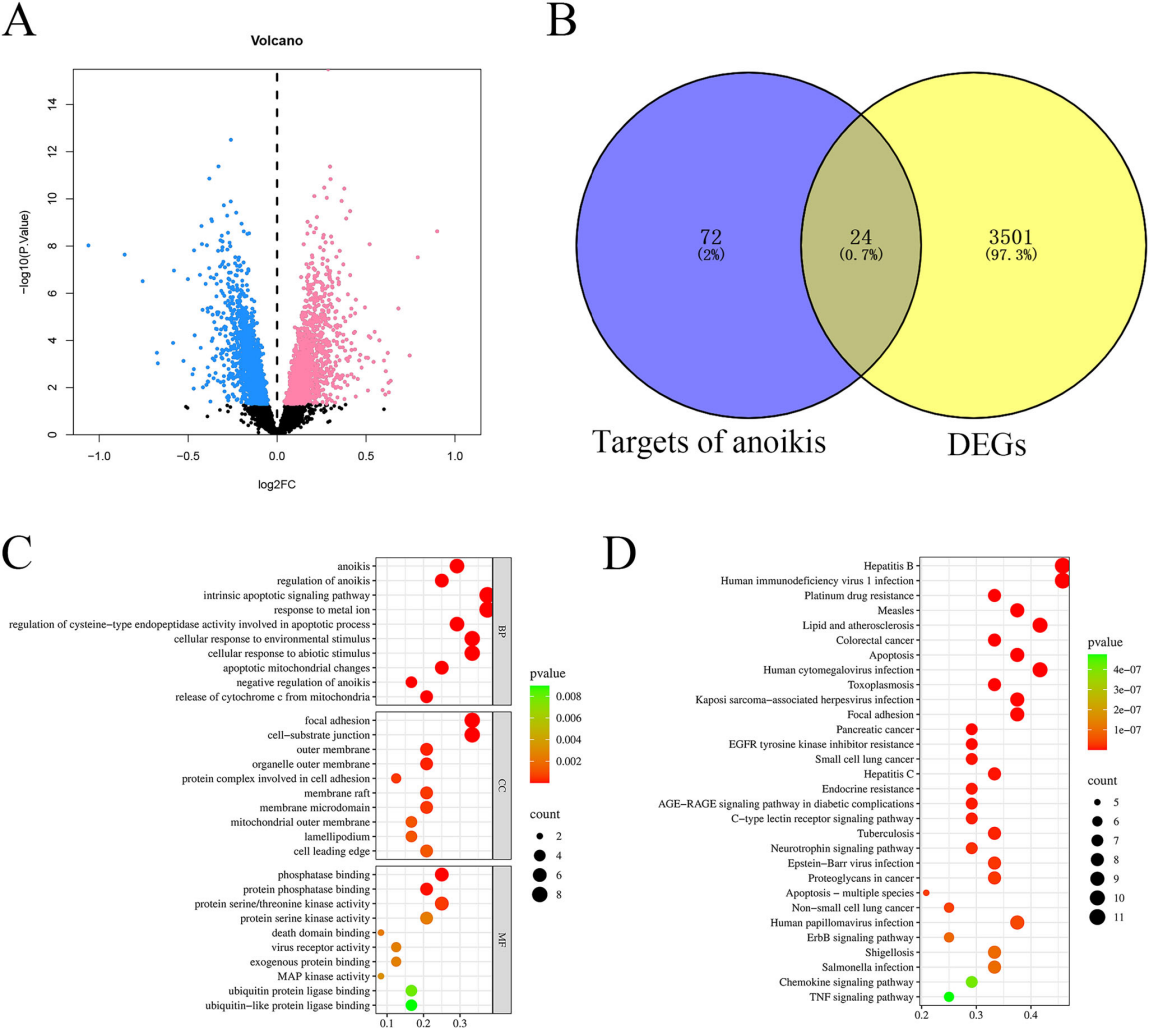
Identification of DEGs in epilepsy. (A) Volcano plot of DEGs, with pink signifying upregulated expression and blue signifying downregulated expression, (B) Venn analysis between DEGs and targets of anoikis, (C) GO enrichment analysis, and (D) KEGG enrichment analysis.

A total of 3525 differential genes were identified from the GSE143272 dataset, consisting of 1652 upregulated and 1873 downregulated genes, and the volcano plot displays an overview of the DEGs (Figure 1A). Then, 96 ARGs were obtained, and 24 intersecting genes were identified by Venn analysis (Figure 1B). GO and KEGG enrichment analysis were used to analyze the pathways associated with these intersecting genes. GO results showed that these genes were mainly enriched in the intrinsic apoptotic signaling pathway and the cellular response to abiotic stimuli (Figure 1C), and KEGG results indicated that these genes exhibited enrichment primarily in the c‐type lectin receptor signaling pathway, neurotrophin signaling pathway, and ErbB signaling pathway (Figure 1D).

### 3.2 Identification of Potential Predictive Markers of Epilepsy Using the LASSO Model

**FIGURE 2:**
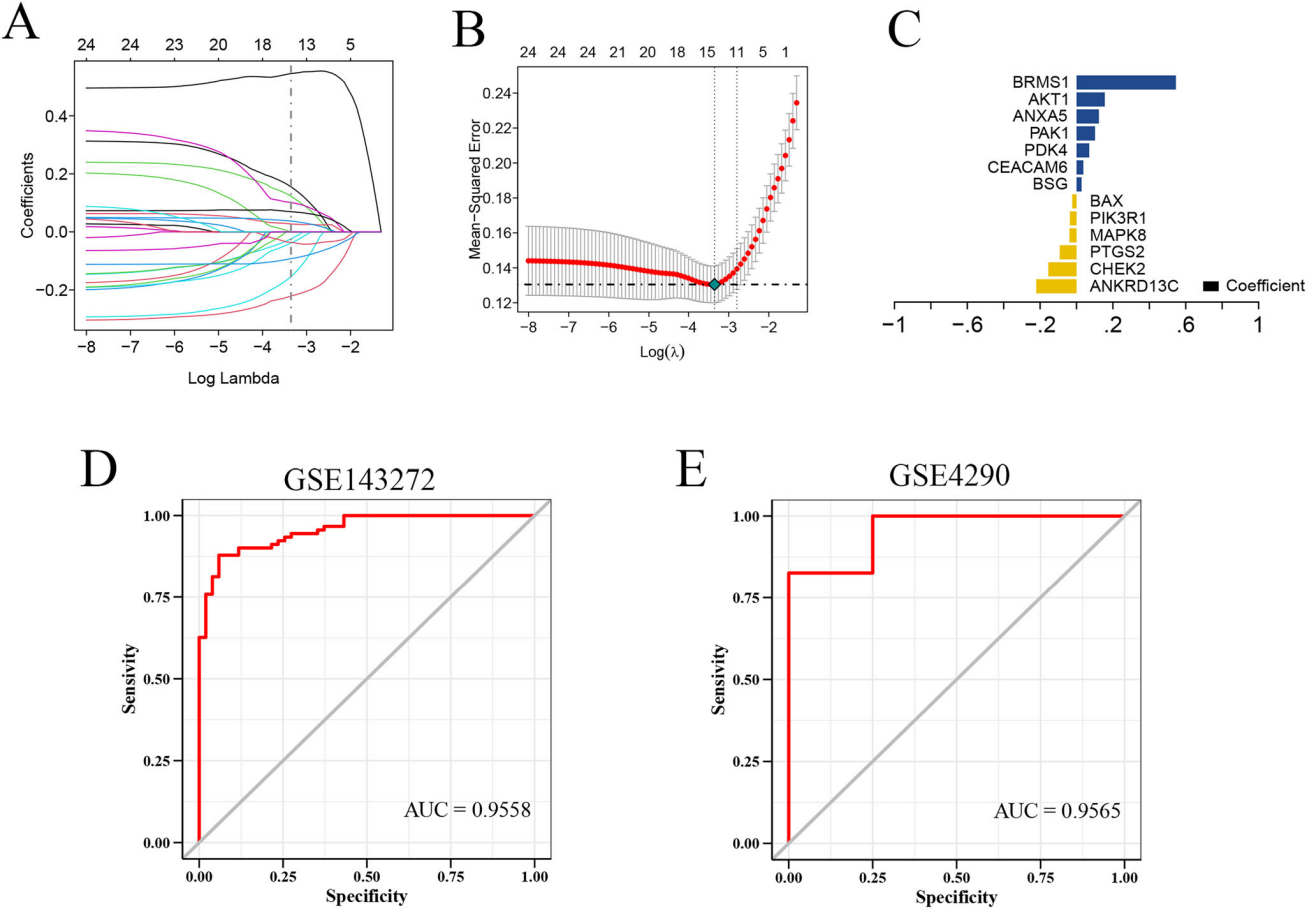
Identification of key genes associated with epilepsy and assessment of the independent predictive performance of the key genes. (A) The distribution of the LASSO coefficients, (B) The confidence intervals for each lambda, (C) Thirteen genes were identified through LASSO regression and their corresponding coefficients, and (D) The ROC curve analysis of the training set. (E) The ROC curve analysis of the GSE4290 validation set.

We utilized the GSE143272 dataset as the training set and the GSE4290 dataset as the validation set and selected intersection genes for feature screening through LASSO regression. LASSO regression consensus identified 13 genes as characteristic genes of epilepsy, namely: ANKRD13C, CHEK2, PTGS2, MAPK8, PIK3R1, BAX, BSG, CEACAM6, PDK4, PAK1, ANXA5, AKT1, and BRMS1. These 13 genes were selected as key genes for subsequent investigations and for establishing a prediction model (Figures 2A–B). The formula for the model is as mentioned: Risk score = ANKRD13C × (−0.221091258475995) + CHEK2 × (−0.154716139704634) + PTGS2 × (−0.092242622389523) + MAPK8 × (−0.0386863939889303) + PIK3R1 × (−0.036274398631166) + BAX × (−0.0239017302044628) + BSG × 0.0274686988581336 + CEACAM6 × 0.0375046859436846 + PDK4 × 0.0697699385826017 + PAK1 × 0.101635869643401 + ANXA5 × 0.122716856514644 + AKT1 × 0.155330006941868 + BRMS1 × 0.545729468062632 (Figure 2C). The outcomes were indicative of the good diagnostic efficiency exhibited by the prediction model based on the 13 genes, exhibiting an area under the curve (AUC) of 0.9558 (Figure 2D). Additionally, we used the GSE4290 dataset as the validation set. Further validation of the diagnostic model was carried out utilizing an external dataset, and the outcomes were indicative of its robust stability, with an AUC of 0.9565 on the GSE4290‐AUC curve (Figure 2E).

### 3.3 WGCNA Analysis and Key Modules in Epilepsy Identified

**FIGURE 3:**
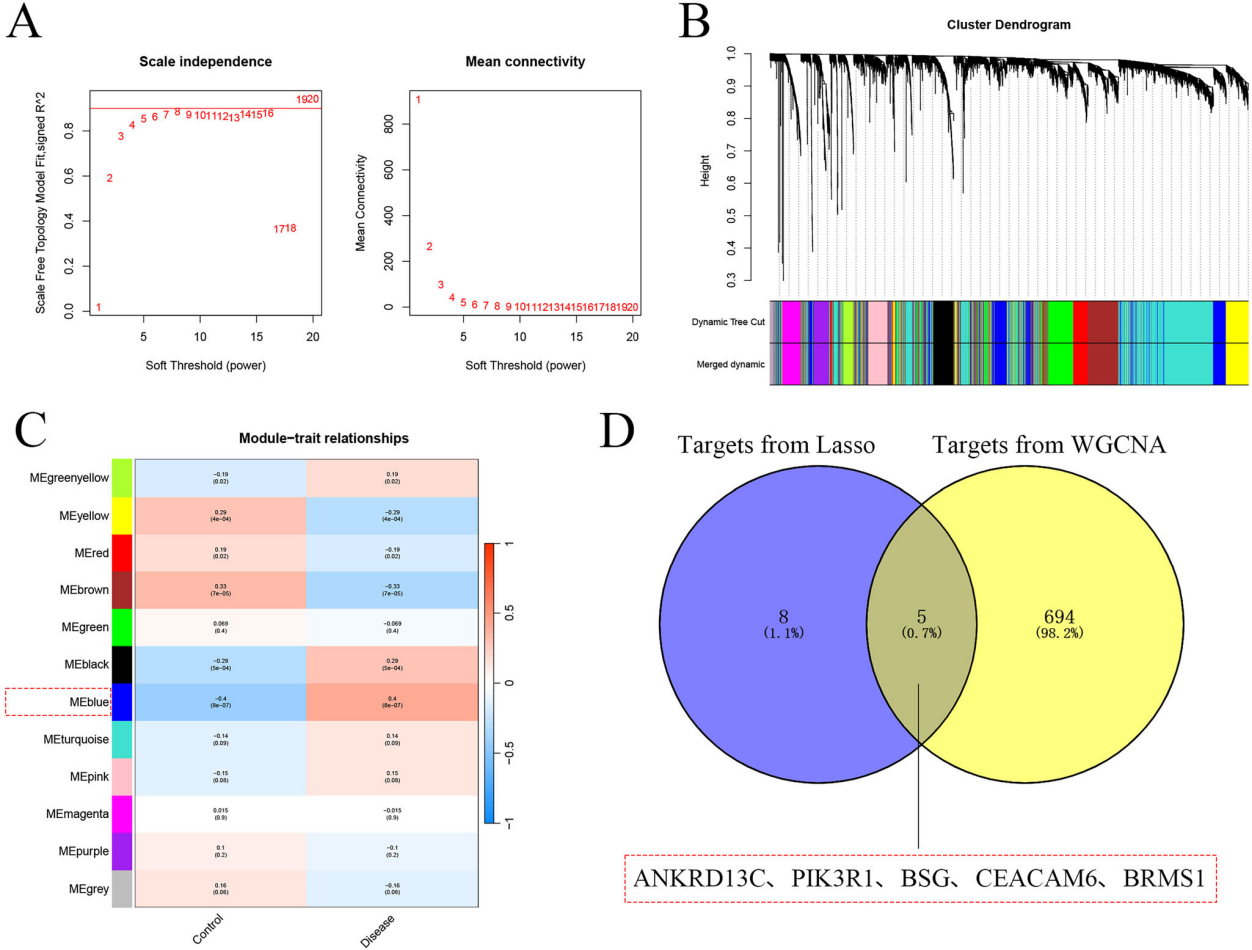
WGCNA analysis and DE‐ARGs identified. (A) Scale‐free fit index and mean connectivity for various soft‐thresholding powers, (B) Dendrogram depicting gene clustering, with unique colors denoting distinct modules, (C) Heatmap showing the association between module eigengenes and epilepsy, with blue indicating negative correlation and red indicating positive correlation, and (D) Venn diagram delineating key genes.

To identify key genes in the epilepsy cohort, we further established the WGCNA network using expression profile data. The soft threshold β was determined using the function “sft$powerEstimate.” Setting the soft threshold to 5 (Figure 3A) enabled the detection of gene modules based on the TOM. Overall, 12 gene modules were identified in this analysis (Figures 3B–C), namely blue (*R* = 0.4, *p* = 8e‐07), black (*R* = 0.29, *p* = 5e‐04), green (*R* = ‐0.069, *p* = 0.4), and others. The blue module demonstrated the highest correlation with the disease, and genes of blue were intersected, with five intersecting genes acquired (ANKRD13C, PIK3R1, BSG, CEACAM6, and BRMS1) (Figure 3D). These five intersecting genes were considered the DE‐ARGs for our subsequent investigation.

### 3.4 Immune Cell Infiltration Analysis

**FIGURE 4:**
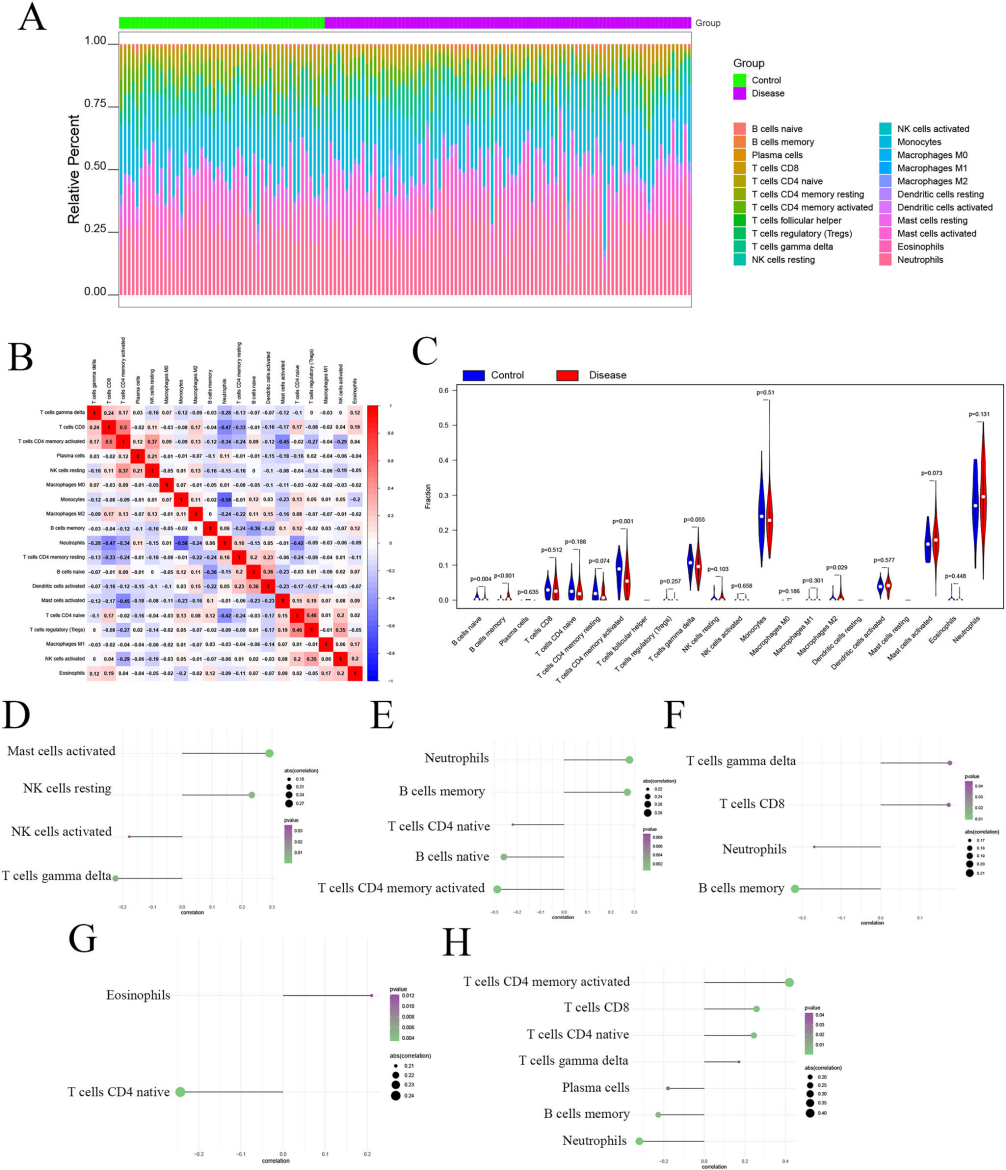
Immune infiltration status. (A) Relative percentages of 22 immune cell subgroups in the samples, (B) Correlations between the 22 immune cell subgroups, with blue indicating positive correlations and red indicating negative correlations, (C) Differences in immune cell content between normal and epileptic samples, with blue representing normal samples and red representing epileptic samples, and (D–H) Correlations between the expression levels of key genes and the content of immune cells. *p < 0.05.

**FIGURE 5:**
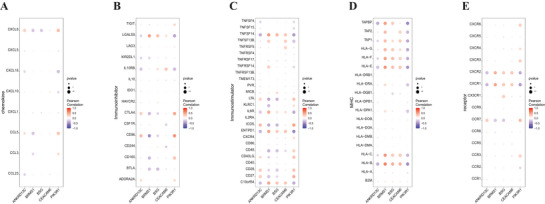
Association between key genes and immune factors. Pearson correlation bubble plots illustrating the correlation between key genes and the following immune factors. (A) Chemokines, (B) Immuno‐inhibitors, (C) Immuno‐stimulators, (D) MHC, and (E) receptors.

The microenvironment predominantly comprises extracellular matrix, immune cells, inflammatory factors, various growth factors, and distinctive physicochemical features, significantly influencing disease diagnosis and responsiveness to clinical treatment. Through an analysis of the correlation between DE‐ARGs and immune infiltration in the epilepsy dataset, we investigated the possible molecular mechanisms via which DE‐ARGs impact the progression of epilepsy. The proportions of immune cell content in every patient and the correlations between immune cells are exhibited in Figures 4A–B. Additionally, we observed significant differences in memory B cells, M2 macrophages, activated CD4 memory T cells, and naive B cells between the two groups (*p* < 0.05) (Figure 4C). The association between DE‐ARGs and immune cells was further assessed. Expectedly, five DE‐ARGs exhibited strong correlations with immune cells (Figures 4D–H). Subsequently, we explored the relationship between these five DE‐ARGs and various immune factors, encompassing chemokines, immunomodulators, and cell receptors, using the TISIDB (Figures 5A–E). These assessments suggested a strong association between DE‐ARGs and the level of immune cell infiltration, indicating their significant function in the immune microenvironment.

### 3.5 Significant Pathways Involved With Key Genes

**FIGURE 6:**
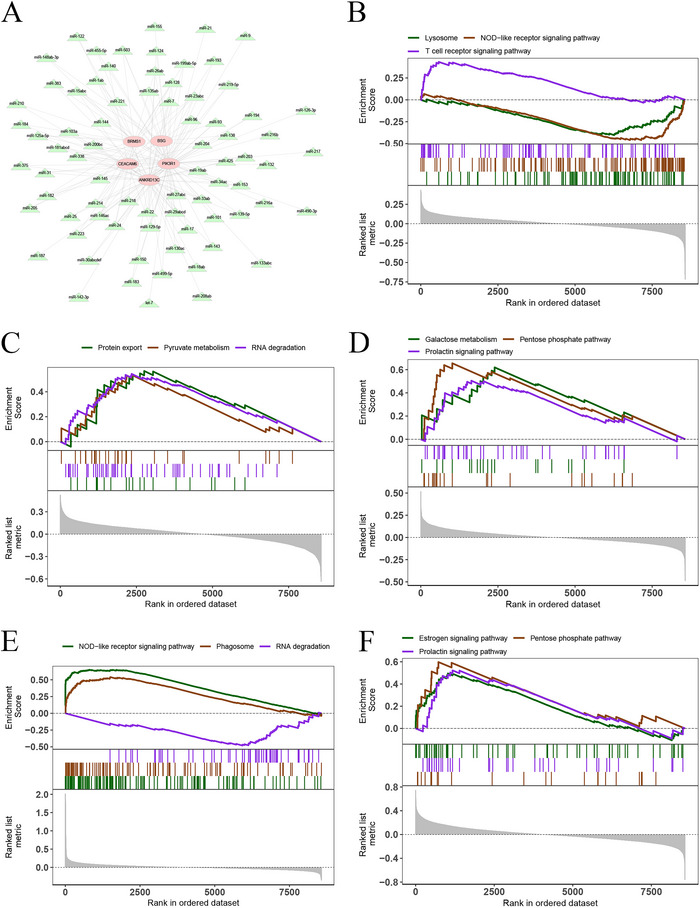
MiRNA network and GSEA of key genes. (A) Visualization of the miRNA network of key genes via Cytoscape software. KEGG signaling pathways involving ANKRD13C (B), PIK3R1 (C), BSG (D), CEACAM6 (E), and BRMS1 (F) along with pathway regulation and associated genes.

The specific signaling pathways linked to the enrichment of the five DE‐ARGs were further assessed for a more in‐depth understanding using the miRcode database, and results revealed 76 miRNAs and 175 mRNA‐miRNA relationships with five DE‐ARGs (Figure 6A). Then, GSEA depicted that ANKRD13C exhibited enrichment in pathways associated with lysosomes, NOD‐like receptor signaling, and T‐cell receptor signaling (Figure 6B and Figure S2A). PIK3R1 exhibited enrichment in protein export, pyruvate metabolism, RNA degradation, and other pathways (Figure 6C and Figure S2B). BSG exhibited enrichment in pathways associated with galactose metabolism, the pentose phosphate pathway, and prolactin signaling (Figure 6D and Figure S2C). CEACAM6 exhibited enrichment in pathways linked to NOD‐like receptor signaling, phagosomes, and RNA degradation, among others (Figure 6E and Figure S2D). BRMS1 exhibited enrichment in pathways associated with estrogen signaling, the pentose phosphate pathway, and prolactin signaling (Figure 6F and Figure S2E).

In addition, GSVA revealed that ANKRD13C exhibited high expression levels in the Myc targets v2, E2F targets, and mTOR1 signaling pathways (Figure S3A). PIK3R1, with a notably high expression, was found to be enriched in the Myc targets v1, P53 pathway, and IL‐2/STAT5 signaling pathways (Figure S3B). BSG demonstrated considerable high expression in the IL‐6/JAK/STAT3 signaling, ROS pathway, and Notch_signaling pathways (Figure S3C). CEACAM6 displayed heightened expression primarily in Notch_signaling, Wnt beta‐catenin_signaling, heme metabolism, and other signaling pathways (Figure S3D). BRMS1 exhibited a considerable high expression in the ROS pathway, PI3K/AKT/mTOR signaling pathway, and IL‐6/JAK/STAT3 signaling pathway (Figure S2E). These results suggest that DE‐ARGs may impact the progression of epilepsy via modulation of these pathways.

### 3.6 scRNA‐seq Profiling of Brain Tissues From Patients With TLE

**FIGURE 7:**
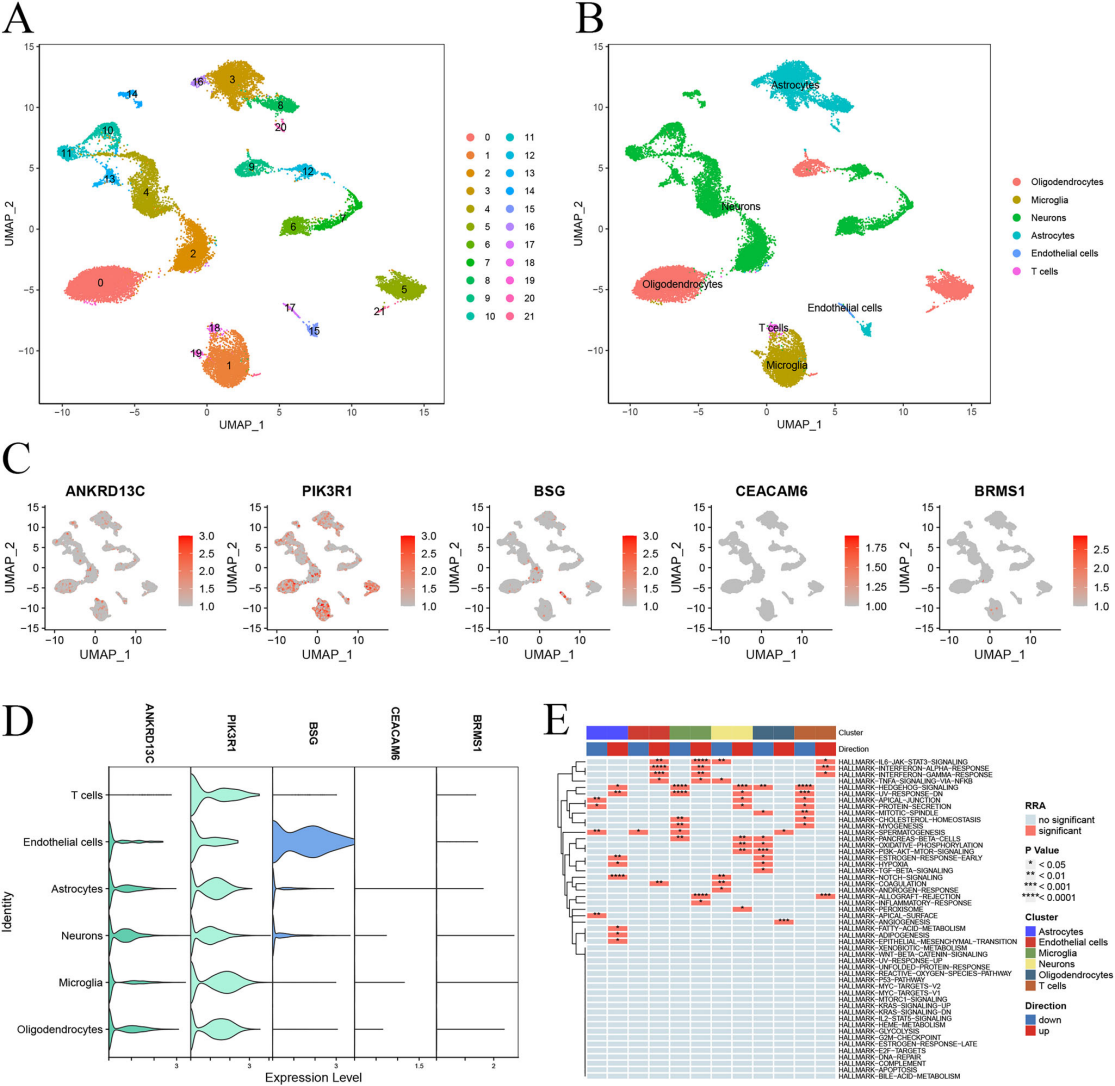
Single‐cell profiling of brain tissue from patients with TLE. (A–B) UMAP plots of identified cells, (C) Normalized expression of DE‐ARGs for each cell type. (D–E) Violin plots and heatmap of DE‐ARGs across six clusters.

We analyzed the scRNA‐seq dataset (GSE190452) derived from the brain tissues of patients with TLE, obtained from the GEO database. After gene expression normalization, which addressed read depth and mitochondrial read count, a subset of 5963 cells was selected from a total of 13,206 cells in patients with TLE. Employing PCA in conjunction with a graph‐based clustering technique, we identified six distinct cell clusters: microglia (characterized by CSF1R and TGFBR1 expression), endothelial cells (marked by FLT1 and ABCB1 expression), astrocytes (identified by SLC1A2 and ADGRV1 expression), oligodendrocytes (marked by TNR and MBP expression), T cells (expressing CD247 and STAT4), and neurons (identified by DLGAP1 and NRGN expression), as depicted in Figures 7A–B. The DE‐ARGs for each cell type were visualized in a UMAP plot (Figure 7C) and further detailed in a violin plot and heatmap (Figures 7D–E).

### 3.7 Experimental Validation of the Biomarker in Epilepsy by qRT‐PCR and Western Blot Assay

**FIGURE 8:**
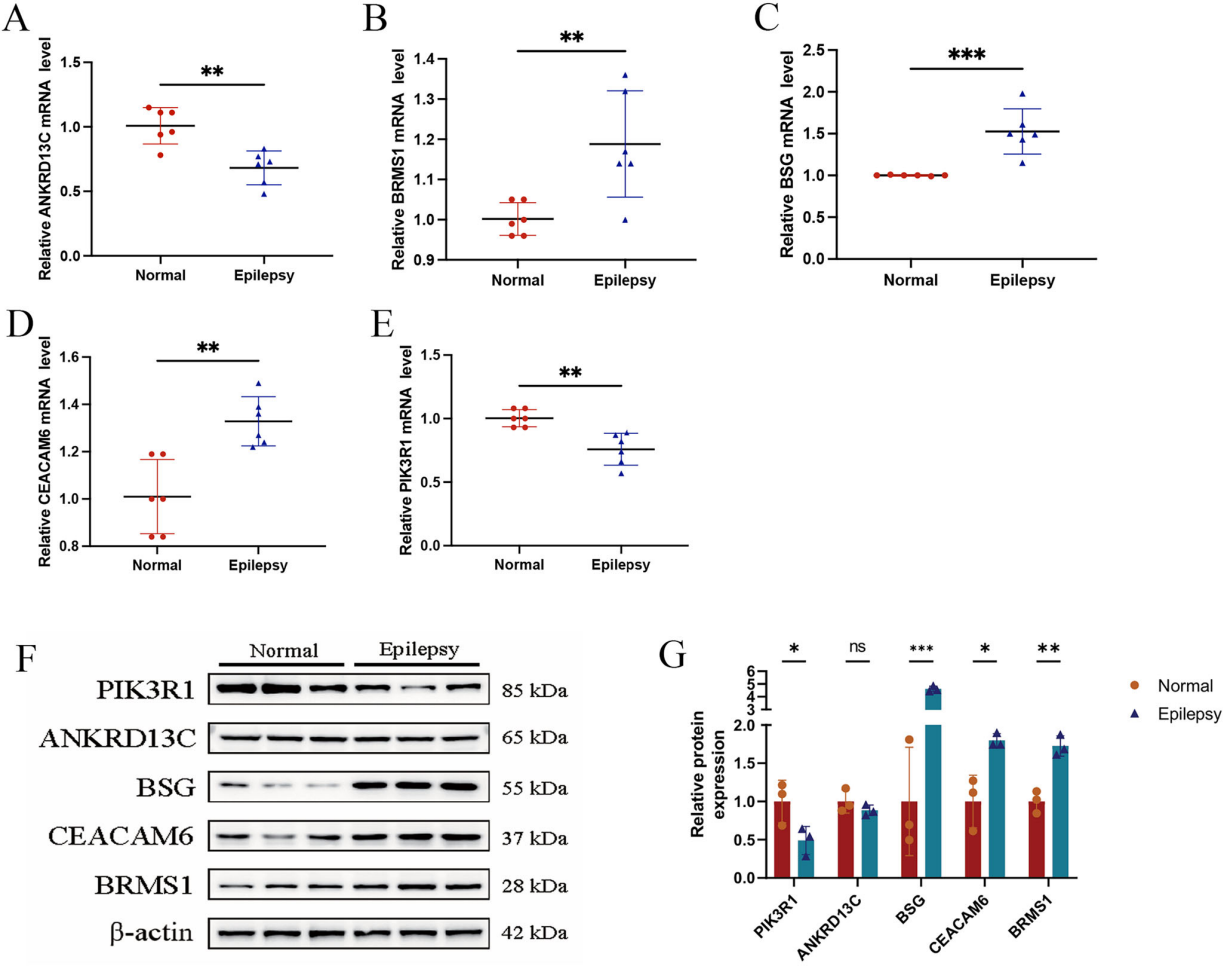
Valuation of key genes in animal models. (A–E) The mRNA expression levels of *ANKRD13C*, *PIK3R1*, *CEACAM6*, *BSG*, and *BRMS1* in the hippocampal tissue of normal and epilepsy models. (F–G) Western blot analysis and relative quantification demonstrate the protein levels of ANKRD13C, PIK3R1, CEACAM6, BSG, and BRMS1 in hippocampal tissues from both normal and epilepsy models. ***p < 0.0005, **p < 0.005, and *p < 0.05; *n* = 6.

To validate whether *ANKRD13C*, *PIK3R1*, *BSG*, *CEACAM6*, and *BRMS1* were expressed as predicted, we conducted experimental validation through qRT‐PCR, utilizing hippocampal tissues obtained from epilepsy mouse models. The qRT‐PCR results are depicted in Figures 8A–E. The acquired data indicated that *ANKRD13C* and *PIK3R1* exhibited lower expression levels in epilepsy hippocampal tissues in contrast to controls (*p* < 0.05, Student's *t*‐test, *n* = 6), while *BSG*, *CEACAM6*, and *BRMS1* were highly expressed. The PIK3R1 protein exhibited decreased expression in the epilepsy group, whereas BSG, CEACAM6, and BRMS1 exhibited increased expression in the same group (Figures 8F–G). Notably, ANKRD13C did not exhibit significant alterations in expression between the normal and epilepsy groups.

### 3.8 Transcriptional Regulation Analysis of DE‐ARGs and Candidate Small Molecules Identified

**FIGURE 9:**
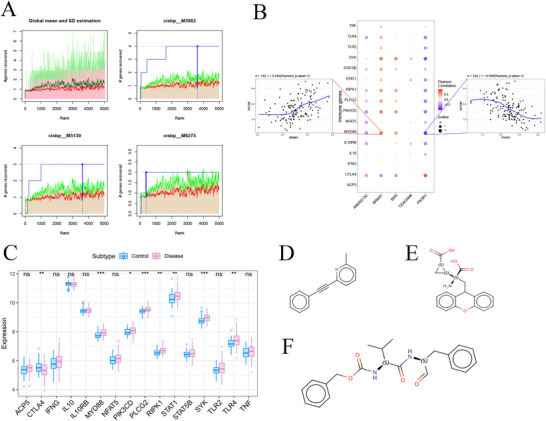
Transcriptional regulation analysis and prediction of potential therapeutic drugs for key genes. (A) Four motifs exhibiting high AUC values. The red line signifies the average recovery curve for every motif, while the green line signifies the average + standard deviation, and the blue line represents the current motif's recovery curve. The highest enrichment level was assessed by identifying the point of maximum distance (mean + standard deviation) between the current motif and the green curve, (B) Differential expression of disease‐regulating genes, with blue signifying controls and pink signifying patients with disease, (C) Pearson correlation analysis of key genes and disease‐associated genes, with blue signifying negative correlation and red signifying positive correlation, and (D–F) The 2D structure diagrams of potential therapeutic drugs predicted via the CMap database.

We applied the five DE‐ARGs to the gene set under analysis and found that they were governed by shared mechanisms, such as those involving multiple transcription factors. Consequently, these transcription factors exhibited enrichment through cumulative recovery curves. Motif‐tf annotation and selection analysis of pivotal genes revealed that the motif cisbpM3802 exhibited the highest standardized enrichment score (NES = 6.94). All enriched motifs and their associated transcription factors for DE‐ARGs are illustrated in Figure 9A. Utilizing the GeneCards database (https://www.genecards.org/), we acquired immune‐linked genes. Analysis of gene expression disparities revealed substantial variation in the expression levels of CTLA4, MYD88, PIK3CD, PLCG2, RIPK1, and STAT1 between the two patient groups (Figure 9B). Subsequently, we conducted a correlation analysis between DE‐ARGs and immune‐linked genes, which demonstrated a significant correlation between the expression levels of DE‐ARGs and those of immune‐linked genes (Figure 9C). Notably, BRMS1 and MYD88 exhibited a significant positive correlation (Pearson *R* = 0.436), while PIK3R1 and MYD88 exhibited a negative correlation (Pearson *R* = −0.508).

In pursuit of a therapeutic strategy, the top 150 upregulated and downregulated genes were categorized into two distinct sets. We predicted potential drugs for these DEGs by the CMap database. The results revealed significant correlations between the expression profiles of the drug disturbances MPEP, LY‐341495, and MDL‐28170 and those of the disease, which may be potential drugs for epilepsy (Figures 9D–F).

## 4 Discussion

While neuronal loss in epilepsy is traditionally attributed to excitotoxicity‐induced apoptosis and ferroptosis, the specific contribution of anoikis—programmed cell death triggered by the loss of cell‐matrix interactions—is gaining increasing attention. Neuronal survival is fundamentally dependent on the structural support of the extracellular matrix (ECM) and the intracellular signaling mediated by the integrin family, which serves as the primary physical link between the cytoskeleton and the ECM (Zhang et al. 2025). Disruption of these connections is not merely a passive consequence of tissue damage but a driver of epileptogenesis. For instance, recent studies have revealed that the physical interaction between Integrin‐α5 and the KCNB1 potassium channel is critical for neocortical development; the dissociation of this complex mimics an anoikis‐like state, leading to neuronal defects and increased seizure susceptibility (Bortolami et al. 2023). Mechanistically, this detachment stress is sensed by “sentinel” proteins such as Bcl‐2 modifying factor (Bmf). As a sensor of cytoskeletal integrity, Bmf is unleashed upon loss of cell adhesion, translocating to mitochondria to initiate the apoptotic cascade (Pervushin et al. 2025). These findings collectively suggest that the disruption of “anchorage” signals creates a pro‐epileptogenic microenvironment. However, the specific gene landscape regulating this anoikis process in the epileptic brain remains to be fully elucidated. The complex nature of epilepsy, a prevalent neurological condition, underscores the necessity for targeted diagnostic and therapeutic strategies. Our study integrated bioinformatics and high‐throughput sequencing to investigate the role of anoikis in epilepsy; results revealed five potential biomarkers and provided data support for the development of new targeted therapeutic strategies for epilepsy. Analysis of the GSE143272 dataset identified 3525 DEGs, Venn analysis initially screened 24 ARGs, and subsequent machine learning and WGCNA analyses identified five key DE‐ARGs genes, namely *ANKRD13C*, *PIK3R1*, *BSG*, *CEACAM6*, and *BRMS1*, which emerged as promising diagnostic markers.

ANKRD13C is a human protein encoded by the *ANKRD13C* gene and belongs to the ankyrin repeat domain‐containing family, with a distinctive protein structure (Tanno et al. 2012). It has been identified as a molecular chaperone for G protein‐coupled receptors (GPCRs), regulating their biogenesis and exit from the endoplasmic reticulum, and thereby modulating downstream signaling (Parent et al. 2010). Moreover, ANKRD13C participates in *Ca* ^2^^+^ dysregulation through GPCR‐activated TRPC channels, which can promote secondary brain injury and may ultimately contribute to epileptogenesis (Parmar et al. 2023). In our scRNA‐seq dataset, ANKRD13C was detectable across all six major cell types, with modest but consistent expression in both neuronal and non‐neuronal clusters. This broad distribution suggests that ANKRD13C may influence epilepsy not only via neuronal excitability but also by shaping immune and glial signaling, particularly in microglia and T‐cell clusters enriched for innate and adaptive immune pathways. Such a pattern is in line with its enrichment in lysosome, NOD‐like receptor, and T cell receptor signaling pathways observed by GSEA and supports the hypothesis that ANKRD13C integrates GPCR trafficking with immune‐related mechanisms in epilepsy, which warrants functional validation in cell‐type–specific models (Parent et al. 2010; Parmar et al. 2023).

*PIK3R1* encodes the regulatory subunit p85 of class I phosphoinositide 3‐kinases (PI3Ks), which are central mediators of cell growth, survival, metabolism, and intracellular trafficking (Huang et al. 2019). PI3K/AKT/mTOR signaling has been implicated in epileptogenesis, and pharmacological inhibition of PI3K shows anticonvulsant effects in experimental models, highlighting this pathway as a potential therapeutic target (Mazumder et al. 2019). Elevated PIK3R1 expression has also been reported in a macaque model of mesial temporal lobe epilepsy, further supporting its involvement in seizure‐related plasticity (Chen et al. 2021). In our scRNA‐seq analysis, PIK3R1 showed broad expression in neurons, astrocytes, oligodendrocytes, microglia, and endothelial cells, consistent with its role as a ubiquitous signaling hub. Neuronal expression is compatible with regulation of survival and synaptic plasticity, whereas robust expression in microglia and astrocytes suggests that PI3K/AKT signaling also shapes neuroinflammatory responses in the epileptic microenvironment. Indeed, PI3K–AKT activation is known to modulate microglial polarization and cytokine production, thereby linking extracellular danger signals to transcriptional programs that influence neuronal damage and network hyperexcitability (Chu et al. 2021). Together, these findings indicate that cell‐type–specific PIK3R1 activity coordinates neuronal survival and glial inflammation, providing a mechanistic bridge between anoikis‐related signaling and epilepsy progression.

*BSG*, also known as basigin, extracellular matrix metalloproteinase inducer, or CD147, is a transmembrane glycoprotein of the immunoglobulin superfamily with diverse physiological and pathological roles (Sameshima et al. 2000). It is expressed in monocytes and macrophages, where it can activate the ERK/NF‐κB pathway through interaction with cyclophilin A, thereby promoting matrix metalloproteinase production and inflammation (Yuan et al. 2010; Kim et al. 2009). Elevated *BSG* levels have been reported in several neuroinflammatory conditions, and inhibition of CD147 can attenuate tissue damage and improve functional outcomes in models of brain injury (Liu et al. 2019; Dang et al. 2015). Our scRNA‐seq data showed that BSG is abundantly expressed in endothelial cells and glial populations, including astrocytes and microglia, but is also present in neurons. This pattern is in keeping with CD147's established roles at the neurovascular interface and in glial activation: endothelial BSG contributes to blood–brain barrier (BBB) function and leukocyte trafficking, whereas glial BSG may modulate cytokine release, extracellular matrix remodeling and metabolic coupling (Wang et al. 2021). In the context of epilepsy, higher BSG expression in microglia, and endothelial cells may therefore facilitate BBB disruption, immune‐cell infiltration, and chronic neuroinflammation, while neuronal expression could influence vulnerability to excitotoxic stress. These cell‐type–specific effects suggest that targeting BSG/CD147 might simultaneously modulate neurovascular integrity and glial activation in epileptic networks.

*CEACAM6*, also known as CD66c, is a member of the carcinoembryonic antigen‐related cell adhesion molecule family and functions as a cell‐surface adhesion protein (Kuespert et al. 2006). Under physiological conditions, CEACAM6 shows low expression in most normal epithelial and hematopoietic cells but is markedly upregulated in many epithelial cancers, where it promotes cell adhesion, invasiveness, and resistance to anoikis (Honda et al. 1997; Schölzel et al. 2000; Kanderová et al. 2010). In our cohort, CEACAM6 was associated by GSEA with NOD‐like receptor signaling, phagosome, and RNA degradation pathways, suggesting a role in immune modulation and cellular stress responses. At the single‐cell level, CEACAM6 transcripts were sparse in resident brain cells and predominantly confined to infiltrating immune clusters, consistent with reports that CEACAM6 is highly expressed on neutrophils and can regulate their adhesion and inflammatory activation (Skubitz 2024). These observations support the interpretation of CEACAM6 as a marker of inflammatory cell recruitment rather than a parenchymal gene in epilepsy, implying that its contribution to epileptogenesis is likely indirect, through facilitating immune‐cell adhesion and persistence within epileptogenic foci.

BRMS1, encoded by the *BRMS1* gene, was initially identified as a metastasis suppressor that reduces the metastatic potential of human breast cancer and melanoma cell lines without affecting primary tumorigenicity (Phadke et al. 2008, Shevde et al. 2002). Mechanistically, BRMS1 functions as a transcriptional corepressor by promoting recruitment of histone deacetylases to the RelA/p65 subunit of NF‐κB, leading to deacetylation of lysine 310 and suppression of NF‐κB‐dependent gene transcription, thereby enhancing apoptosis and limiting pro‐inflammatory signaling (Liu et al. 2006). In our study, BRMS1 was enriched in pathways related to estrogen signaling, the pentose phosphate pathway, and prolactin signaling, indicating a possible link to hormonal and metabolic regulation in epilepsy. scRNA‐seq analysis revealed moderate BRMS1 expression across neurons, astrocytes, oligodendrocytes, and microglia, suggesting that its NF‐κB‐repressive activity may operate in multiple cellular compartments. In glial cells, higher BRMS1 levels could constrain excessive inflammatory gene expression and thereby dampen neuroinflammation, whereas in neurons, BRMS1 may modulate stress‐responsive transcriptional programs and apoptosis susceptibility. Given the central role of NF‐κB in seizure‐induced inflammation and neuronal injury, these cell‐type–specific patterns support the view that BRMS1 acts as a shared negative regulator of pro‐inflammatory pathways in the epileptic brain and may represent a potential target to fine‐tune neuroinflammatory responses without globally suppressing immune function.

The role of the immune microenvironment in neurological conditions, including epilepsy, is increasingly being acknowledged. Our research highlighted considerable variations in specific immune cell populations—memory B cells, M2 macrophages, activated CD4 memory T cells, and naive B cells—between patients with epilepsy and healthy individuals. These disparities provide insights into the underlying mechanisms of epilepsy, warranting further investigation. Additionally, the CMap database was employed to identify three potential therapeutic drugs for epilepsy, including MPEP, LY‐341495, and MDL‐28170. MPEP acts as a selective and non‐competitive antagonist of a subtype of glutamate receptors (Gass and Olive 2008), and plays a crucial role in numerous neurotransmitter systems, such as epilepsy (Sanon et al. 2010). A study indicated that MPEP can reduce neuronal excitability to control or reduce epileptic seizures (Mares et al. 2010). However, MPEP's efficacy appears context‐dependent, varying with seizure type and developmental stage. In one study, MPEP failed to affect spike‐and‐wave discharges (a signature of absence seizures) in adolescent rats, even though it strongly suppressed electrically induced convulsive seizures in younger animals (Lojková and Mares 2005). Besides, LY‐341495 is a selective antagonist of mGluRs, especially mGluR2 and mGluR3 subtypes, which play a role in regulating neurotransmitter release and neuronal excitability (Seo et al. 2022; Folbergrová et al. 2005). LY‐341495 can inhibit these receptors to reduce the frequency of spike‐wave discharges in the rat model of absence epilepsy (Ngomba et al. 2005). Notably, in the WAG/Rij rat model of genetic absence epilepsy, systemic LY‐341495 dose‐dependently reduced the frequency of spontaneous spike‐wave discharges, the hallmark of absence seizures (Ngomba et al. 2005). MDL‐28170, also known as calpain inhibitor III, is a potent, cell‐permeable, and selective inhibitor of calpain, a family of calcium–dependent cysteine proteases (Urthaler et al. 1997). Epileptic seizures can elevate intracellular calcium levels by upregulating calpain, resulting in excessive neuronal discharge, thus leading to neurodegeneration and epilepsy (Araújo et al. 2008). Previous studies show that MDL‐28170 demonstrated efficacy in reducing seizure severity and frequency in the TLE model (Lam et al. 2017). But overall, although these drugs show promise for the treatment of epilepsy, these compounds have not yet been widely applied in clinical practice for epilepsy treatment.

Despite these strengths, several limitations should be acknowledged. First, although we used a pilocarpine‐induced epilepsy model and evaluated seizure severity with the Racine scale, we did not perform EEG recordings. Thus, behavioral scoring alone may miss subclinical or electrographic seizures; future studies will incorporate EEG monitoring to more rigorously validate the model. Second, the GSE4290 dataset used for external validation has a small number of control samples, which might limit statistical power. Third, our findings were not validated in an independent clinical cohort beyond the public datasets. Future work will focus on larger independent patient cohorts to validate the DE‐ARG signatures and risk model across diverse populations. Finally, although we confirmed the differential expression of the five DE‐ARGs in vivo, we did not directly measure anoikis events (cell detachment‐induced death) in neurons or glia. Future experiments will in‐depth explore molecular mechanisms of these anoikis genes in epilepsy.

## 5 Conclusions

This study integrated multiple methods to screen out five key DE‐ARGs, including *ANKRD13C*, *PIK3R1*, *BSG*, *CEACAM6*, and *BRMS1*, involved in the pathogenesis of epilepsy. These DE‐ARGs are linked to epilepsy by affecting the immune system. In summary, our work provides new insights and data support for the research of epilepsy and the development of more therapeutic strategies.

## Author Contributions

Conceptualization, HZ and YY; methodology, WZ; validation, HZ; formal analysis, WZ and XC; investigation, HZ, YY, TT and XC; resources, XZ and XC; data curation, WZ and TT; writing – original draft preparation, HZ and YY; writing – review and editing, XZ and XC; visualization, YY, XC and WZ; supervision, XZ; project administration, WZ and XZ; funding acquisition, XZ and YY. All authors have read and agreed to the published version of the manuscript.

## Ethics Statement

All animal experiments conducted in this study were approved by the Institutional Animal Care and Use Committee (IACUC) of Southern Medical University and were carried out in strict accordance with the ethical guidelines of the National Institutes of Health Guide for the Care and Use of Laboratory Animals (Grant No.20210405006).

## Conflicts of Interest

The authors declare no conflicts of interest.

## Supporting information




**Supplementary Materials**: brb371273‐sup‐0001‐SuppMat.docx

## Data Availability

The data presented in this study are openly available in Gene Expression Omnibus (GEO) (URL: https://www.ncbi.nlm.nih.gov/geo/, accessed on January 17, 2023).
