# Supplementary material for: Identification and Verification of Anoikis‐related Genes in Epilepsy Through Bioinformatics Analysis
Source: Brain Behav. 2026 Feb 16;16(2):e71273. doi: 10.1002/brb3.71273 (PMC12910123; doi:10.1002/brb3.71273)
Supplement: Supplementary file 1 — Supplementary Materials: brb371273‐sup‐0001‐SuppMat.docx [file BRB3-16-e71273-s001.docx]

**Table S1. Primer sequences**

| ANKRD13C Forward | GCAGCAACCCCGCTTTAGT |
| --- | --- |
| ANKRD13C Reverse | GGATGAGCGAGGACAGTCTC |
| PIK3R1 Forward | ACACCACGGTTTGGACTATGG |
| PIK3R1 Reverse | GGCTACAGTAGTGGGCTTGG |
| BSG Forward | GGCTGGTTTCCTCAAGGCA |
| BSG Reverse | TAGGCGGCATGGATGTGAAC |
| CEACAM6 Forward | TCAATGGGACGTTCCAGCAAT |
| CEACAM6 Reverse | CACTCCAATCGTGATGCCGA |
| BRMS1 Forward | TGAACGGGGAGGCAGATGA |
| BRMS1 Reverse | GTTCCCTGAACAACTTCTCCTTC |
| ADAM17 Forward | TTGTCTCCAAATGCCGTCTG |
| ADAM17 Reverse | CTTCTTACAGCACTTGGCTTTC |

**Table S2. Antibodies**

|  | Dilutability | Company | Antibody batch numbers |
| --- | --- | --- | --- |
| ANKRD13C | 1:2000 | Abmart | RY110379 |
| PIK3R1 | 1:2000 | Abmart | Q63787 |
| BSG | 1:2000 | Abcam | ab188190 |
| CEACAM6 | 1:2000 | Abmart | B3Y547 |
| BRMS1 | 1:2000 | Abmart | Q5M7T3 |
| β-actin | 1:3000 | Abcam | ab6276 |

**Figure 1s**


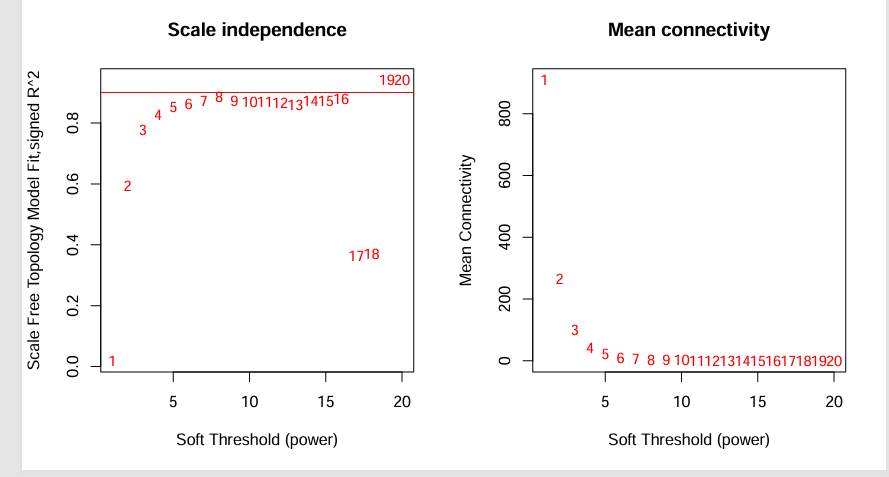


**Figure S2. GSEA**

**
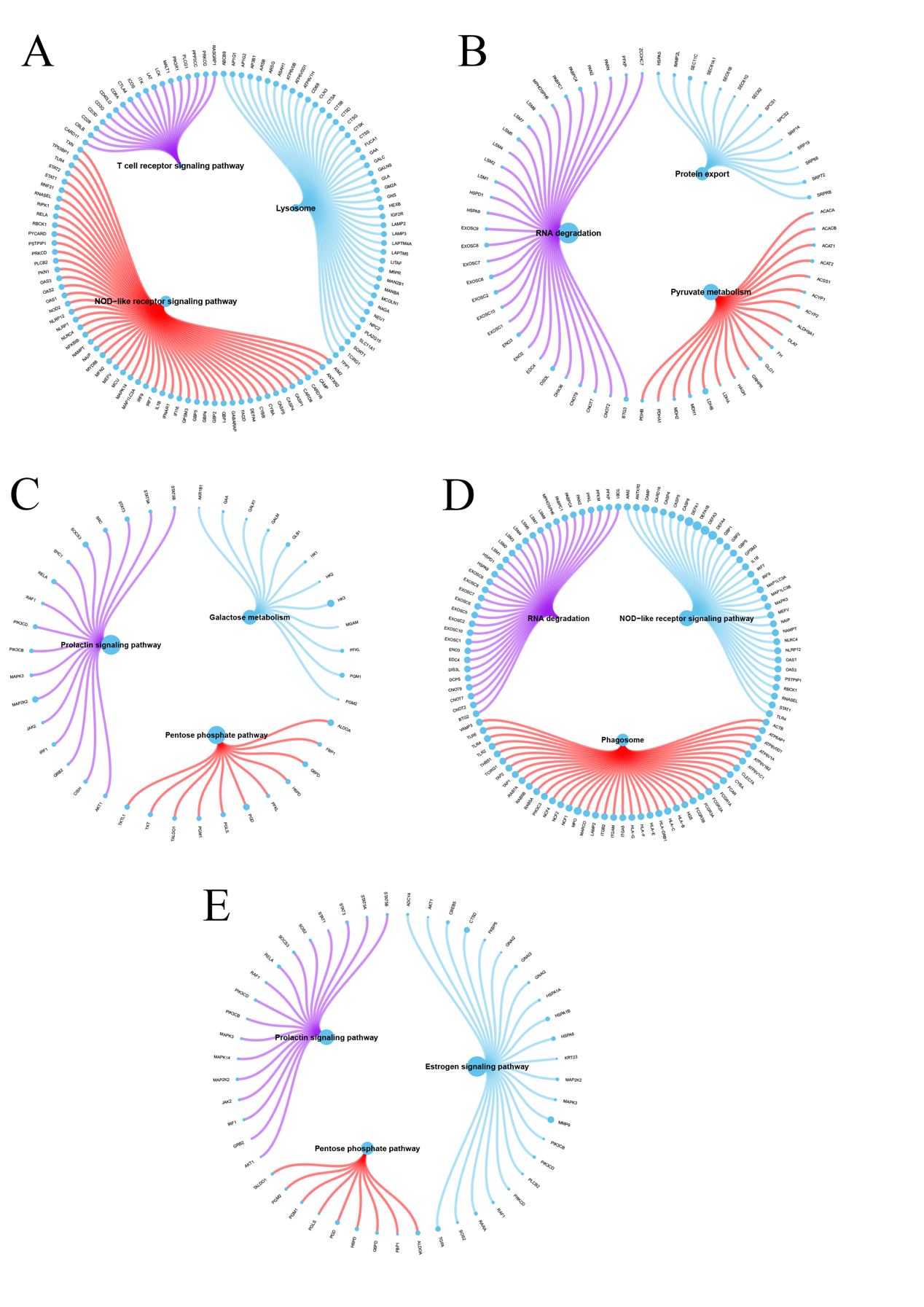
**

**Figure S3 GSVA**

**
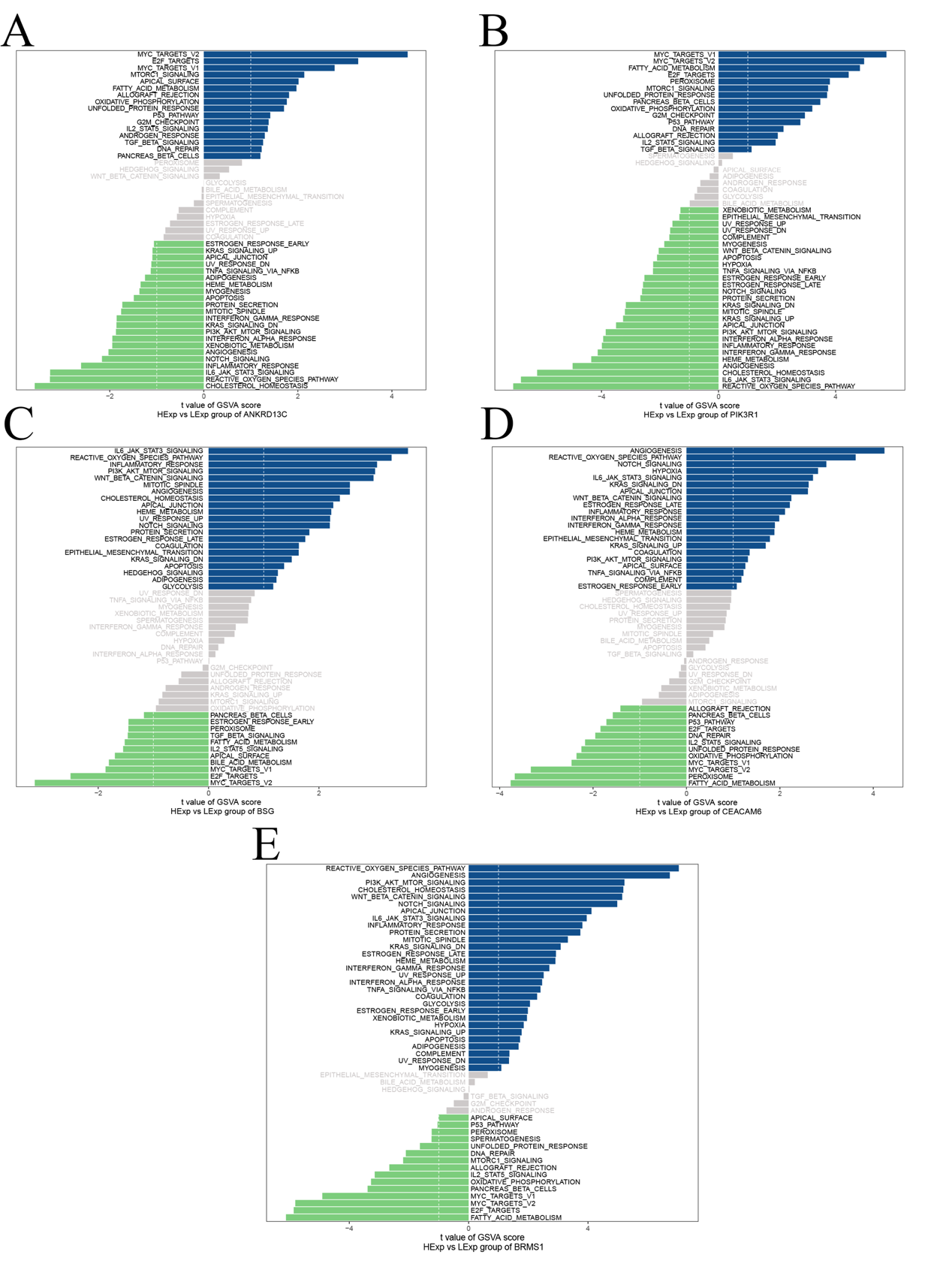
**
